# Paravertebral Block and Perioperative Ketamine in an Opioid-Sparing Analgesia Approach in Video-Assisted Thoracic Surgery: A Retrospective Single-Centre Study

**DOI:** 10.3390/jcm14165765

**Published:** 2025-08-14

**Authors:** Daniele Marianello, Francesco Ginetti, Filippo Sanfilippo, Cesare Biuzzi, Chiara Catelli, Elena Modica, Francesca Silva, Alessandra Cartocci, Luca Luzzi, Roberto Corzani, Piero Paladini, Sabino Scolletta, Federico Franchi

**Affiliations:** 1 Department of Medical Science, Surgery and Neurosciences, Cardiothoracic and Vascular Anesthesia and Intensive Care Unit, University Hospital of Siena, 53100 Siena, Italy; daniele.marianello@unisi.it (D.M.); francesco.ginetti89@gmail.com (F.G.); elena@modica.me (E.M.); f.silva@student.unisi.it (F.S.); 2 Department of Anaesthesia and Intensive Care, A.O.U. Policlinico-San Marco, 95123 Catania, Italy; filipposanfi@yahoo.it; 3 Department of Medicine, Surgery and Neurosciences, Urgency-Emergency Anaesthesia and Intensive Care Unit, University Hospital of Siena, 53100 Siena, Italy; cesare.biuzzi@ao-siena.toscana.it (C.B.); sabino.scolletta@unisi.it (S.S.); 4 Thoracic Surgery Unit, University Hospital of Siena, 53100 Siena, Italy; chiara.catelli@ao-siena.toscana.it (C.C.); luca.luzzi@unisi.it (L.L.); robcorzani@gmail.com (R.C.); piero.paladini@unisi.it (P.P.); 5 Department of Medical Sciences, Surgery and Neurosciences, University of Siena, 53100 Siena, Italy; alessandra.cartocci@dbm.unisi.it

**Keywords:** chronic pain, morphine consumption, locoregional anaesthesia, video assisted thoracoscopy

## Abstract

**Background**: Regional anaesthesia techniques allow postoperative pain control while reducing opioid consumption. Ketamine is another viable option for minimising perioperative opioid use. We evaluated the efficacy of a perioperative multimodal analgesia protocol incorporating paravertebral block (PVB) and ketamine infusion in patients undergoing video-assisted thoracic surgery (VATS). **Methods**: This retrospective single-centre study divided patients into two groups: the opioid-sparing (OS) group receiving PVB and ketamine (n = 41), and the control group (n = 21) treated with postoperative morphine infusion. The primary outcome was the need for rescue opioid therapy; secondary outcomes included postoperative pain scores assessed at multiple time points over 48 h using the numeric rating scale (NRS), prevalence of chronic postoperative pain at three months, perioperative haemodynamics, and hospital length of stay. **Results**: Rescue opioid administration was significantly lower in the OS group (19.5% vs. 47.6%, *p* = 0.021). Upon awakening, pain control was better in the OS group (1 [1–2] vs. 4 [3–4], *p* < 0.001); however, pain scores did not differ afterwards. Chronic postoperative pain was less common in the OS group (n = 10/41; 23.8% vs. n = 11/21, 52.4%; *p* = 0.028). No differences in haemodynamics were reported, nor were there any ketamine/PVB-related complications. No difference in length of hospital stay was observed between the groups. The ketamine starting dose and postoperative morphine requirements were inversely correlated (rho = −0.380; *p* = 0.002). **Conclusions**: A multimodal analgesia protocol integrating PVB and ketamine infusion in patients undergoing VATS may effectively reduce postoperative opioid consumption, improving analgesia in the initial postoperative period.

## 1. Introduction

Patients undergoing thoracic surgery frequently experience severe acute postoperative pain (APOP), even after minimally invasive surgical approaches such as video-assisted thoracic surgery (VATS) [1]. Inadequate pain control may lead to delayed recovery, impaired respiratory function, and a higher incidence of postoperative complications, including infections, and prolonged hospital stays with increased costs [2,3,4]. Opioids have been a mainstay of APOP management after major surgery, with administration ranging from intravenous to transdermal [5]. However, their use can be associated with adverse effects, such as nausea, vomiting, constipation, sedation, delayed mobilisation, and respiratory depression [5,6]; hence, there is a constant search for alternatives in the management of APOP, aimed at enhancing recovery and minimising side effects. In this context, APOP can be effectively controlled by multimodal analgesia approaches [3,4,5]. Multimodal analgesia refers to a variety of strategies aimed at optimising analgesia through the combination of different pharmacological and regional techniques. Such combinations aspire to achieve synergistic effects while minimising systemic opioid consumption and its inevitable side effects [3,4,5,6].

Regional anaesthesia techniques include thoracic epidural anaesthesia (TEA), which has historically been the gold standard for reducing opioid consumption in the management of APOP in thoracic surgery. However, TEA is now less frequently used due to the development of ultrasound-guided thoracic wall blocks, which provide similar outcomes with the advantage of being less invasive. The development of ultrasound-guided thoracic wall blocks has gained interest and wide clinical application [7,8], even in the setting of cardiac surgery [9]. Among the thoracic wall blocks, the paravertebral block (PVB) may offer advantages over other regional anaesthesia techniques. Indeed, compared to the erector spine or serratus anterior plane blocks, PVB targets the origin of the intercostal nerves and provides unilateral analgesia by blocking somatic and sympathetic nerves already in the paravertebral space, making it particularly effective for unilateral thoracic procedures [10,11,12,13]. As part of enhanced recovery after surgery (ERAS) protocols for thoracic procedures, PVB seems to offer comparable analgesia to TEA while reducing the risk of opioid-related respiratory complications [14], contributing to a smoother recovery process [15,16,17]. Recent ERAS guidelines encourage the use of regional anaesthesia techniques to improve postoperative outcomes and reduce opioid consumption [14,16,17]. Conversely, an aspect that requires further research is the implementation of opioid-sparing (OS) multimodal analgesia protocols in which regional anaesthesia is combined with adjuvant drugs such as ketamine [17,18,19,20].

In this retrospective study, we aimed to assess the efficacy of a multimodal analgesia protocol combining ultrasound-guided thoracic PVB and perioperative intravenous ketamine infusion in reducing postoperative opioid consumption and improving pain control in patients undergoing thoracic surgery.

## 2. Materials and Methods

### 2.1. Study Population

This retrospective study utilised prospectively collected data as part of an internal audit on the management of APOP in patients undergoing VATS between March and October 2020 at the Thoracic Surgery Unit of the University Hospital of Siena. This means that clinical data were recorded in real time as part of routine practice, without any protocolised data collection driven by research purposes. No prospective study design or intervention was applied. The study was approved by the local ethics committee (Protocol n. 18862, approved on 15 March 2021). Data collection began on 1 September 2022, and data analysis was performed starting from 1 June 2023.

Our audit included all adult patients undergoing VATS lung resection. We excluded patients younger than 18 years, pregnant individuals, those requiring postoperative intensive care unit monitoring, re-do surgeries or emergency procedures, those with known allergies to local anaesthetics, and individuals with a history of neuro-psychiatric conditions that could preclude accurate pain assessment. Patients receiving chronic opioid therapy or regular non-steroidal anti-inflammatory drugs before surgery were also excluded to avoid confounding effects on postoperative analgesia.

Patients were grouped according to their usage of OS, particularly in terms of intraoperative PVB and ketamine infusion (case group), or the intra- and postoperative use of opioids (control group). General anaesthesia induction was identical in both groups and was performed in all patients with propofol (1–3 mg/kg), fentanyl (1–2 mcg/kg), and rocuronium (0.5 mg/kg) boluses. Endotracheal intubation was achieved with a double-lumen tube (Robertshaw type), and intraoperatively, general anaesthesia was maintained in all patients with sevoflurane (1.0–1.5 minimum alveolar concentration). The control group received intraoperative fentanyl boluses (0.1–0.3 mcg/kg every 30 to 45 min). Conversely, the OS group did not receive intraoperative opioids (apart from the induction of anaesthesia) and was managed with PVB and ketamine. Specifically, the thoracic PVB was performed before inducing general anaesthesia with the patient in the lateral decubitus position [21]. The T4 paravertebral space was identified using a high-frequency (6–18 MHz) linear ultrasound probe (Epiq 5-Philips^®^ Eindhoven, Amsterdam, the Netherlands) with an in-plane approach, placing the probe parallel to the vertebral column [22]. The block consisted of a single injection of 0.5% ropivacaine, titrated based on the patient’s ideal body weight (0.1 mL/kg or a maximum of 15 mL total volume), using a 100 mm nerve block needle (Stimuplex A’ B. Braun). At the end of surgery, sedation was discontinued, and patients were extubated upon being neurologically appropriate and demonstrating adequate spontaneous ventilation.

### 2.2. Postoperative Analgesia

At the end of surgery, both groups received intravenous paracetamol (1 g) and ketorolac (30 mg), which were also administered perioperatively at regular intervals. Specifically, intravenous paracetamol was administered at doses of 1 g every 8 h, and ketorolac 30 mg was given every 12 h, except in cases of contraindications (allergies, chronic kidney disease stage > 2, or a history of gastrointestinal bleeding). Apart from the use of paracetamol and ketorolac (common to both arms), the control group received intravenous morphine (starting dose of 0.1 mg/kg followed by an infusion of 0.2 mg/kg over 24 h for 30 h), while the OS group received a starting dose of ketamine (variable dose of 0.20–0.25 mg/kg) just before extubation, followed by continuous infusion at a dose of 20 mcg/kg/h for 30 h via an elastomeric pump. This dose falls within the sub-dissociative analgesic range, and no emergence phenomena, such as hallucinations, dissociation, or delirium, were observed in any patient.

Whenever the nurse’s assessment of analgesia revealed uncontrolled pain, every 8 h, (defined as numeric rating scale (NRS) > 4), a rescue analgesic dose (oral morphine 10 mg) was administered. The ward nurse on duty was unaware of the contents of the elastomeric pump (ketamine or morphine), or of the execution of PVB. Assignment to one of the two groups was related to the ability or otherwise of the anaesthetist on duty to perform the PVB.

### 2.3. Data Collection and Analysis

Demographic characteristics, including age, sex, BMI, and ASA classification, were recorded for all patients. Additionally, detailed perioperative data were gathered, encompassing the type and duration of surgery, haemodynamic parameters (systolic, diastolic, and mean blood pressure, SpO2, and heart rate), and the administration of anaesthetic and analgesic drugs. Postoperative pain scores were assessed using the NRS and documented immediately after extubation and subsequently at six other postoperative time points (every 8 h for 48 h). The need for rescue opioid therapy (intravenous morphine 0.01 mg/kg) was also recorded, along with any reported complications (such as postoperative nausea and vomiting–PONV, respiratory depression, or itching) and the total length of hospital stay (LOS). In the immediate postoperative period, morphine was administered intravenously (0.01 mg/kg) if needed, whereas in the ward, oral morphine (10 mg) was used as rescue analgesia according to the standard protocol.

The primary outcome was the incidence of the rescue dose of oral morphine (10 mg), which was administered when the NRS score was above 4. The assessment according to the NRS and the administration of rescue doses were by a nurse blinded to the intraoperative management to ensure an unbiased evaluation. The secondary objectives included (1) postoperative pain intensity (NRS), recorded immediately after surgery and then every 8 h up to 48 h; (2) the development of chronic postoperative pain, which was assessed three months later via telephone interview; (3) the differences in oxygen saturation (SpO2) and blood pressure; and (4) the LOS.

### 2.4. Statistical Analysis

A minimum sample size of 59 patients was estimated considering a Fisher exact test, with the first type error set at 0.05 and a power of 85%, using an estimated relative difference in rescue opioid dose administration between groups of 30% with a between-group ratio of 1:2. This ratio was determined according to the local availability of anaesthesiologists rotating in the thoracic surgery theatre and their ability to provide PVB and willingness to use ketamine infusion in the context of an OS approach. Power analyses were performed using G*Power (Erdfelder, Faul & Buchner 2020, version 3.1.9.7). Categorical variables were summarised using absolute numbers and percentage frequencies, while quantitative variables were summarised using median and interquartile range as the Kolmogorov–Smirnov test showed that the distribution of data was not normal. Hence, we used the non-parametric Mann–Whitney tests. Differences between categorical variables were tested with Fisher’s exact test. A *p*-value of <0.05 was considered statistically significant. Analyses were carried out with R (The R Foundation for Statistical Computing 2023, version 4.3.2).

## 3. Results

### 3.1. Study Population

A total of 62 consecutive patients were included in the study, 39% of whom were male (n = 24). Patients underwent VATS lung resection, specifically enrolling 55 lobectomies and seven segmentectomies. Among the enrolled patients, 41 were assigned to the OS group and 21 to the control group. Descriptive preoperative and perioperative characteristics are presented in Table 1. No differences were observed between groups in the intraoperative parameters, and no complications directly attributable to the PVB or the administration of analgesic drugs during the perioperative period were reported.

### 3.2. Outcome

As shown in Table 2, the incidence of rescue oral morphine administration within 48 h after surgery was significantly lower in the OS group (n = 8/41, 19.5%) compared to the control group (n = 10/21, 47.6%, *p* = 0.021). Specifically, 8 doses were given to 8 patients in the OS group (one rescue each), while 22 doses were given to the 10 patients in the control group (8 patients received 2 doses, and 2 patients received 3 doses of rescue analgesia; *p* < 0.001). Considering the overall number of APOP evaluations (seven per patient), the occurrence of rescue dose administrations was significantly lower in the OS group (n = 8/287 vs. 22/147, *p* < 0.0001). A post hoc exploratory analysis was conducted according to the different starting doses of ketamine and showed a negative correlation between the ketamine starter dose and the need for rescue oral morphine (Spearman’s Rho = −0.380; *p* = 0.002).

**Figure 1:**
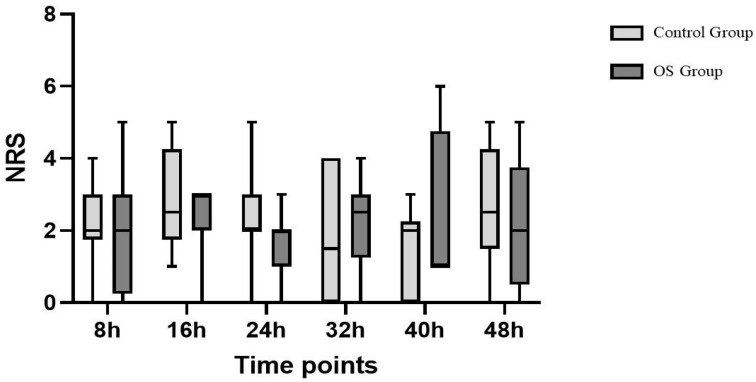
NRS. NRS after surgery was lower in OS group (1 [1–2]) compared to control group (4 [3–4]; *p* > 0.001).

Patients in the OS group reported significantly lower NRS scores upon awakening (Table 2 and Figure 1), but no statistically significant differences in NRS scores were detected at later time points (from the 8th until the 48th postoperative hour) with median values consistently ranging between 1 and 2 points of the NRS (Table 2).

Upon telephone interview three months after surgery, chronic postoperative pain was reported with significantly lower prevalence in the OS group (n = 10/41, 23.8%) compared to the control group (n = 11/21, 53.4%, *p* = 0.028; Table 2).

We found no differences in haemodynamic values and SpO2, apart from higher SpO2 levels and lower heart rate at awakening in the OS group. Similarly, there was no statistically significant difference in the hospital LOS between the two groups (OS group vs. control group 5 [5–8] vs. 6 [5–7] days; *p* = 0.541) (Table 3).

No opioid-related adverse events, such as nausea, vomiting, pruritus, constipation, sedation, urinary retention, respiratory depression, or delirium, were observed in either group. There were no statistically significant differences between groups in the occurrence of these complications. Similarly, no respiratory complications were reported after thoracic surgery in either group.

## 4. Discussion

In this retrospective study, we report data from an internal audit of patients undergoing VATS with the aim of evaluating the potential benefits of an OS approach. We applied a multimodal analgesia protocol that included a thoracic PVB and an OS analgesia regimen with intravenous ketamine supported by paracetamol and ketorolac at the end of the surgical procedure.

Our findings support the hypothesis of clinical benefits from our multimodal intervention with PVB and ketamine. Indeed, this approach significantly reduced the need for postoperative rescue doses (oral morphine); interestingly, we also noted a possible dose-dependent effect of the ketamine starting dose on opioid sparing, with a greater initial dose correlated to a lower number of rescue doses. The latest ERAS guidelines in the context of lung surgery recommend a standardised multimodal pain management strategy incorporating locoregional anaesthesia to minimise postoperative opioid consumption [16]. Reducing opioid use is crucial due to the well-documented adverse effects, including PONV, paralytic ileus, excessive sedation, and prolonged hospital stays [23,24]. Additionally, multimodal analgesia incorporating locoregional techniques shortens the duration of postoperative mechanical ventilation, facilitates early mobilisation and physiotherapy, and reduces the risk of atelectasis and pneumonia [25]. Our results align with the available evidence, adding scientific information on the value of combining a locoregional anaesthesia approach (PVB in our cohort) with ketamine. Our study demonstrates that multimodal analgesia incorporating both PVB and ketamine might be superior to an opioid-based protocol, with improved pain scores on awakening and reduced rescue doses, although the NRS scores between groups were not significantly different at later time points [26]. These findings suggest that a multimodal analgesic approach combining paravertebral block and perioperative ketamine may contribute to improved early postoperative pain control and reduced opioid use, particularly during the immediate recovery phase, in line with current ERAS recommendations for thoracic surgery [1,12,14]. This finding highlights the potential of enhancing OS strategies not only with the implementation of locoregional anaesthesia but also with the addition of intravenous ketamine to achieve comparable pain relief while mitigating opioid-related side effects.

Ketamine, an N-methyl-D-aspartate (NMDA) receptor antagonist, is also a known and effective adjuvant for analgesia in the perioperative period, reducing opioid use and preventing opioid tolerance and central sensitisation to nociceptive signalling [23,24,25,26,27]. However, its optimal dosing regimen remains controversial [23], and the drug is not easy to handle due to its possible side effects. Suzuki and colleagues reported reduced pain at three months postoperatively when ketamine was combined with preoperative TEA [28], whereas Dualè et al. found improvements only in immediate postoperative pain, with no long-term benefits [29]. The OS effect of ketamine observed in our study aligns with the existing literature and current ERAS recommendations for thoracic surgery. Chumbley et al. conducted a randomised study on 70 patients undergoing thoracotomy, comparing a ketamine infusion (0.1 mg/kg/min preceded by a 0.1 mg/kg bolus) with a control group receiving a placebo. Patients in both groups received TEA, patient-controlled analgesia with morphine, or PVB infusion. Opioid use and numeric pain scores at 24 and 48 h were significantly lower in the ketamine group [24], suggesting its positive modulation of APOP. Furthermore, Jouguelet et al. reviewed the available literature, concluding that five meta-analyses and 39 clinical trials were published on this topic with a total of 2482 patients enrolled, 1403 of whom received ketamine [30]. Their summary of the evidence indicated that among the studies reporting a dose of ketamine similar to that of our approach, six reported a significant reduction in APOP and in opioid consumption within the first 24 h.

Importantly, unlike previous studies, we did not find evidence of ketamine-related side effects, such as hypertension, tachycardia, hallucinations, or delirium. This finding highlights that ketamine could be well tolerated in the context of a multimodal analgesia protocol, reinforcing its potential as an adjuvant and OS strategy.

According to our study protocol, ketamine infusion was preceded by an ultrasound-guided PVB immediately after the induction of general anaesthesia [31,32]. As emphasised by ERAS guidelines, regional anaesthesia is strongly recommended to reduce postoperative opioid use, with PVB providing analgesia equivalent to TEA, particularly for VATS [16,31,32]. Numerous studies have compared PVB and TEA for thoracic surgery pain management. In a recent narrative review, Hamilton et al. concluded that these techniques provide equivalent analgesia for thoracotomy, although TEA could still be considered the gold standard [25]. However, in the case of VATS, the APOP is of a lower degree, and PVB is preferred as it offers comparable pain relief with fewer perioperative complications. A randomised study by Turhan et al. compared the erector spinae plane block, PVB, and intercostal nerve block after VATS. The authors found that all three techniques provided adequate analgesia, though PVB demonstrated superior pain control and reduced morphine consumption [32].

Interestingly, we observed a significant, albeit weak, inverse correlation between the ketamine starting dose and the need for postoperative rescue morphine (*p* = 0.002). Indeed, patients receiving a higher initial dose of ketamine (0.25 mg/kg) exhibited superior pain control compared to those receiving (0.20 mg/kg).

In our study, patients in both groups remained haemodynamically stable throughout surgery, with no need for vasopressors or advanced haemodynamic monitoring, and a lower heart rate at awakening may be the result of better-controlled APOP. Overall, it seems that the PVB did not induce clinically relevant sympatholytic effects. Additionally, no complications related to the locoregional technique (e.g., bleeding, pneumothorax, LAST syndrome, inadvertent intrathecal or intravenous local anaesthetic injection) were observed.

An interesting finding in our study was the reduced incidence of chronic pain at three months after surgery, with the incidence more than halved in the OS group (23.8% compared to 52.4%). This finding further highlights the value of reducing exposure to perioperative opioids, which is a hot topic in anaesthesia. The incidence of chronic pain after thoracic surgery varies between 29% and 35% of patients [33,34]. Yan et al. studied 159 patients undergoing VATS and showed that the opioid-free group (in which a postoperative analgesic block was performed) had a reduced incidence of chronic pain compared to the group in which anaesthesia was conducted with opioids only [35]. However, in a meta-analysis pooling data on 1018 patients, Na et al. did not find a decrease in chronic pain in patients who received PVB or other types of thoracic wall blocks compared to those treated without a block [36]. We had similar findings to Yan et al., but it should be noted that in our study, the intervention group received PVB before surgery, which was associated with ketamine infusion. We believe our encouraging results may be partly due to the execution of PVB before the surgical stimulation, reducing neuronal firing and nociceptive activation following surgical stress [33,37]. Although our study was not primarily designed to assess chronic postoperative pain, the significantly lower prevalence observed in the OS group suggests a potential role for multimodal opioid-sparing strategies in mitigating the development of chronic pain. This is consistent with previous studies highlighting the contribution of regional anaesthesia and ketamine in modulating central sensitisation mechanisms involved in persistent postsurgical pain [22,35].

Our study has several limitations that should be acknowledged. First, it was conducted in a single-centre setting, which may limit the generalisability of the findings to other institutions with different perioperative management protocols. Second, although we prospectively collected data as part of an audit, its retrospective nature introduces potential selection and information biases, which could affect the robustness of our conclusions. Although this was a retrospective study, data were prospectively collected as part of a structured internal audit. This approach may have reduced information bias and improved the consistency and completeness of the dataset. Third, the sample size was limited and unbalanced with a ratio of 2:1. This limitation does not allow the detection of smaller positive effects from the protocol (i.e., reduction in APOP at time points after awakening) or complications related to ketamine or PVB. Larger, multicentre studies would be necessary to confirm our results and offer external validity. Fourth, our study evaluated chronic postoperative pain over a longer timeframe with only a telephone interview, without a structured questionnaire that may be more appropriate for assessing the development of chronic post-thoracotomy pain syndrome. We believe that future studies should incorporate longer follow-up periods and adequate assessment of chronic postsurgical pain to determine whether the sustained benefits of ketamine and regional anaesthesia extend beyond the immediate postoperative phase. Fifth, while we compared a multimodal OS protocol (PVB and ketamine) to a conventional opioid-based regimen, we did not include control groups with the use of each of the two components of our protocol. This exclusion limits our ability to distinguish the specific contributions of the two strategies in reducing opioid consumption. Sixth, the prolonged morphine infusion (48 h) used in the control group may have led to the development of acute opioid tolerance or opioid-induced hyperalgesia, thereby reducing the effectiveness of rescue oral morphine and potentially increasing its consumption. Finally, we did not use a validated questionnaire to assess chronic postoperative pain. Instead, we collected information through a structured telephone interview using targeted questions. While this approach provided basic insights into the persistence and intensity of pain, the lack of a standardised assessment tool may have limited the precision and comparability of these findings. Future studies should consider the use of validated instruments, such as the Brief Pain Inventory or DN4, to ensure more robust assessment of chronic postsurgical pain.

## 5. Conclusions

A multimodal analgesia protocol integrating ultrasound-guided PVB and perioperative ketamine infusion may reduce the need for postoperative rescue doses of opioids while providing effective pain control in the context of an OS approach and reducing the incidence of chronic pain. Such an approach aligns with the ERAS guidelines for OS strategies in thoracic surgery. However, larger randomised studies are needed to determine the clinical benefits of this association and the incidence of ketamine-related side effects.

## Figures and Tables

**Table 1:** Characteristics of the study population. Results are presented as median and interquartile range, and absolute numbers with percentages.

Variables	Overall Population (n = 62)	OS Group (n = 41)	Control Group (n = 21)	*p*
Age, years	68 [64–73]	71 [65–74]	67 [61–73]	0.16
Sex (M), N (%)	24 (39)	18 (44)	6 (29)	0.25
Weight, kg	70 [58–79]	70 [60–80]	65 [55–70]	0.13
Height, cm	166 [160–172]	166 [162–180]	165 [159–175]	0.60
ASA Score	2 [2–2]	2 [2–2]	[2–2]	0.07
Surgery Time, min	136 [117–154]	127 [110–148]	142 [125–155]	0.29

ASA, American Society of Anesthesiologists; OS: opioid sparing.

**Table 2:** Differences in morphine rescue dose use and NRS scale trend in the two groups during the first 48 h after surgery. Results are presented as median and interquartile range, and absolute numbers with percentages.

Variables	Overall Population (n = 62)	OS Group (n = 41)	Control Group (n = 21)	*p*
Morphine rescue (by 48 h)	18 (29%)	8 (19.5%)	10 (47.6%)	0.021
NRS after surgery	2 [1–3]	1 [1–2]	4 [3–4]	<0.001
NRS 8 h	2 [1–2]	2 [1–2]	2 [1–2]	0.66
NRS 16 h	2 [1–3]	1 [1–2]	2 [1–2]	0.50
NRS 24 h	2 [1–2]	2 [1–2]	2 [1–3]	0.24
NRS 32 h	1 [1–2]	0 [0–2]	2 [1–3]	0.12
NRS 40 h	1 [0–2]	1 [1–2]	1 [0–2]	0.37
NRS 48 h	1 [0–2]	1 [0–2]	0 [0–3]	0.11
Chronic pain, n (%)	21 (33.9%)	10 (23.8%)	11 (52.4%)	0.028

NRS, numeric range scale; OS group, opioid-sparing group.

**Table 3:** Haemodynamics and respiratory parameters during the time of observation between the two groups. Results are presented as median and interquartile range.

Variables	Overall Population (n = 62)	OS Group (n = 41)	Control Group (n = 21)	*p*
SBP after surgery, mmHg	120 [110–135]	120 [110–135]	120 [115–140]	0.28
SBP 8 h	130 [120–140]	130 [120–140]	130 [115–140]	0.71
SBP 16 h	125 [115–135]	123 [115–135]	125 [112–135]	0.80
SBP 24 h	125 [115–145]	130 [115–150]	125 [110–130]	0.11
SBP 32 h	125 [116–140]	130 [120–140]	120 [110–130]	0.06
SBP 40 h	125 [115–145]	125 [115–145]	120 [115–125]	0.58
SBP 48 h	130 [120–145]	130 [120–150]	133 [115–140]	0.54
DBP after surgery, mmHg	70 [61–80]	70 [60–80]	70 [65–75]	0.83
DBP 8 h, mmHg	75 [70–85]	80 [70–85]	75 [60–80]	0.26
DBP 16 h, mmHg	75 [65–83]	80 [65–83]	70 [65–80]	0.64
DPB 24 h, mmHg	70 [65–80]	75 [65–85]	70 [60–80]	0.07
DBP 32 h, mmHg	72 [65–80]	75 [68–87]	70 [65–75]	0.15
DBP 40 h, mmHg	75 [65–85]	75 [67–90]	75 [65–80]	0.24
DBP 48 h, mmHg	80 [65–85]	80 [65–90]	75 [67–80]	0.13
HR after surgery, bpm	75 [68–80]	70 [67–75]	85 [76–86]	<0.001
HR 8 h, bpm	74 [70–78]	75 [70–79]	74 [67–76]	0.32
HR 16 h, bpm	75 [70–80]	76 [68–81]	75 [72–80]	0.87
HR 24 h, bpm	78 [70–85]	78 [68–85]	78 [75–84]	0.67
HR 32 h, bpm	74 [70–78]	74 [70–80]	75 [70–78]	0.93
HR 40 h, bpm	77 [70–80]	77 [70–80]	77 [70–80]	0.64
HR 48 h, bpm	78 [70–83]	76 [70–80]	80 [76–90]	0.11
SpO2 after surgery, %	95 [94–97]	95 [94–97]	95 [94–95]	0.02
SpO2 8 h, %	96 [94–97]	96 [94–97]	95 [94–96]	0.20
SpO2 16 h, %	96 [94–97]	96 [94–97]	96 [94–97]	0.96
SpO2 24 h, %	95 [93–97]	96 [93–98]	95 [93–96]	0.12
SpO2 32 h, %	95 [93–97]	95 [94–97]	94 [92–96]	0.14
SpO2 40 h, %	95 [92–96]	95 [93–96]	93 [91–96]	0.18
SpO2 48 h, %	94 [93–96]	94 [93–96]	94 [91–96]	0.29

SBP, systolic blood pressure; DPB, diastolic blood pressure; HR, heart rate; bpm, beats per minute.

## Data Availability

The original contributions presented in this study are included in the article. Further inquiries can be directed to the corresponding author(s).

## Abbreviations

The following abbreviations are used in this manuscript:

APOP	Acute Postoperative Pain
ERAS	Enhanced Recovery After Surgery
VATS	Video-Assisted Thoracic Surgery
TEA	Thoracic Epidural Anaesthesia
PVB	Paravertebral Block
OS	Opioid-Sparing
MHz	Mega Hertz
mg	Milligram
kg	Kilogram
NRS	Numeric Rating Scale
BMI	Body Mass Index
PONV	Postoperative Nausea and Vomiting
LOS	Length Of Hospital Stay
SpO_2_	Blood Oxygen Saturation
ASA	American Society of Anesthesiologists
SBP	Systolic Blood Pressure
DBP	Diastolic Blood Pressure
HR	Heart Rate
bpm	beats per minute
NMDA	Ketamine, an N-methyl-D-aspartate
LAST	Local Anesthetic Systemic Toxicity
